# Supplementary material for: Genomic Insights into the Molecular Basis of Broad Host Adaptability of the Entomopathogenic Fungus Conidiobolus coronatus (Entomophthoromycotina)
Source: J Fungi (Basel). 2025 Aug 19;11(8):600. doi: 10.3390/jof11080600 (PMC12387763; doi:10.3390/jof11080600)
Supplement: Supplementary file 1 [file jof-11-00600-s001.zip › jof-3761496-supplementary.pdf]

Table S1 CCS Reads Data Volume Statistics of *Conidiobolus coronatus.*

Data Type	Sequence Count	Total Base Count	Data N50 Length	Mean Read Length	Maximum Read Length
CCS	425,217	3.840471661	10,712	9,032	30,111

Table S2 Statistics of genome assembly results.

Contig Length (bp)	Contig Number	Contig N50 (bp)	Contig N90 (bp)	GC content (%)	Gaps Number
44,218,739	77	1,415,773	755,109	27.65	0

Table S3 Comparison results statistics.

Library	Mapped(%)	Properly mapped(%)	Coverage(%)	Depth(X)
350bp	99.02	94.32	99.91	82.44

* Library: next-generation sequencing library size; Mapped (%): the percentage of clean reads mapped to the reference genome out of all clean reads; Properly mapped(%): the length distribution of paired-end sequencing sequences that are mapped to the reference genome and are in line with the sequencing fragments; Coverage(%): genome coverage; Depth (X): The depth of genome coverage.

Table S4 BUSCO Evaluation Statistics.

Complete BUSCOs(C)	Complete and single-copy BUSCOs(S)	Complete and duplicated BUSCOs(D)	Fragmented BUSCOs(F)	Missing BUSCOs(M)	Total Lineage BUSCOs
271 (93.45%)	260 (89.66%)	11 (3.79%)	6 (2.07%)	13 (4.48%)	290

* Complete BUSCOs: Find the complete number of genes; Complete and single-copy BUSCOs: the number of single-copy genes; Complete and duplicated BUSCOs: multi-copy gene number; Fragmented BUSCOs: predict the number of incomplete genes; Missing BUSCOs: the number of genes that are not predicted; Total Lineage BUSCOs: Number of fungal conserved gene sets.

Table S5 Statistics of repeat sequence prediction results.

Type	Number	Length (bp)	Percentage (%)
ClassI	10,116	3,988,445	9.02
ClassI/DIRS	304	343,360	0.78
ClassI/LINE	39	48,483	0.11
ClassI/LTR	2	131	0
ClassI/LTR/Copia	33	3,891	0.01
ClassI/LTR/Gypsy	3,777	2,176,500	4.92
ClassI/PLE|LARD	5,938	1,557,245	3.52
ClassI/TRIM	20	16,708	0.04
ClassI/Unknown	3	1,977	0
ClassII	7,211	1,746,618	3.95
ClassII/Helitron	1	40	0
ClassII/MITE	3,021	714,128	1.61
ClassII/Maverick	1,750	360,240	0.81
ClassII/TIR	2,434	787,072	1.78
ClassII/Unknown	5	378	0
PotentialHostGene	3,128	1,334,982	3.02
SSR	111	35,233	0.08
Unknown	4,521	1,462,712	3.31
Total	20,566	7,745,087	17.52

Table S6 Statistics on genetic prediction results.

Method	Software	Species	Gene number
Ab initio based	Augustus		8,605
	Genscan		6,129
	GeneID		5,738
	GlimmerHMM		11,405
	SNAP		13,367
Homology based	GeMoMa	*Conidiobolus coronatus*	11,499
		Entomophthora muscae	6,183
		Neoconidiobolus thromboides	5,954
Integration	EVM		11,128

* Method: gene prediction method; Software: Predictive software; Species: homologous species; Gene number: The number of genes.

Table S7 Basic genetic information statistics.

GeneNum	Genelen	AveGenlen	ExonLen	AveExonLen	ExonNum	AveExonNum	CDSLen
11,128	15,007,320	1,348.61	12,692,259	400.84	31,664	2.85	12,692,259
	AveCDSlen	CDSNum	AveCDSNum	IntronLen	AveIntronLen	IntronNum	AveIntronnum
	400.84	31,664	2.85	2,315,061	112.73	20,536	1.85

Table S8 Gene function annotation statistics.

Database	Number
GO	3,702
KEGG	3,742
KOG	5,571
Pfam	7,176
Swissprot	5,718
TrEMBL	7,397
Nr	7,485
All	8,025

Table S9 Comparison of GO functional annotations between *C. coronatus* and *C. obscurus.*

Functions	*C. coronatus*	Number	*C. obscurus*	Number
cellular component	extracellular region	16	extracellular region	9
	cell	2146	cell	690
	nucleoid	8	nucleoid	1
	membrane	673	membrane	234
	virion	11	virion	0
	cell junction	5	cell junction	6
	membrane-enclosed lumen	151	membrane-enclosed lumen	29
	macromolecular complex	641	macromolecular complex	277
	organelle	1442	organelle	440
	extracellular region part	2	extracellular region part	0
	organelle part	770	organelle part	163
	virion part	11	virion part	0
	membrane part	317	membrane part	77
	cell part	2205	cell part	703
	supramolecular complex	14	supramolecular complex	4
molecular function	transcription factor activity, protein binding	15	transcription factor activity, protein binding	1
	nucleic acid binding transcription factor activity	42	nucleic acid binding transcription factor activity	19
	catalytic activity	1765	catalytic activity	1339
	signal transducer activity	18	signal transducer activity	13
	structural molecule activity	113	structural molecule activity	103
	transporter activity	162	transporter activity	57
	binding	1265	binding	906
	electron carrier activity	36	electron carrier activity	18
	antioxidant activity	15	antioxidant activity	8
	metallochaperone activity	2	metallochaperone activity	0

	protein tag	4	protein tag	0
	molecular transducer activity	2	molecular transducer activity	3
	molecular function regulator	34	molecular function regulator	7
biological process	reproduction	83	reproduction	41
	immune system process	1	immune system process	3
	metabolic process	1801	metabolic process	1074
	cellular process	2039	cellular process	1028
	reproductive process	79	reproductive process	38
	biological adhesion	3	biological adhesion	5
	signaling	74	signaling	51
	multicellular organismal process	22	multicellular organismal process	40
	developmental process	75	developmental process	81
	growth	37	growth	23
	single-organism process	1140	single-organism process	589
	response to stimulus	301	response to stimulus	176
	localization	556	localization	197
	multi-organism process	35	multi-organism process	17
	biological regulation	574	biological regulation	188
	cellular component organization or biogenesis	546	cellular component organization or biogenesis	138
	detoxification	15	detoxification	8
	biological phase	0	biological phase	2
Integrated GO annotated genes		3702		2375

Table S10 KEGG Functional Annotation Statistics of *C. coronatus* and *C. obscurus*.

Functions	*C. coronatus*	Number	*C. obscurus*	Number
Environmental Information Processing	MAPK signaling pathway - yeast	35	MAPK signaling pathway - yeast	53
	ABC transporters	0	ABC transporters	28
	Phosphatidylinositol signaling system	0	Phosphatidylinositol signaling system	31
Cellular Processes	Cell cycle - yeast	77	Cell cycle - yeast	96
	Meiosis - yeast	61	Meiosis - yeast	62
	Endocytosis	58	Endocytosis	74
	Peroxisome	62	Peroxisome	77
	Phagosome	44	Phagosome	62
Metabolism	Alanine, aspartate and glutamate metabolism	29	Alanine, aspartate and glutamate metabolism	36
	Arginine and proline metabolism	46	Arginine and proline metabolism	33
	Cysteine and methionine metabolism	45	Cysteine and methionine metabolism	45
	Glycine, serine and threonine metabolism	47	Glycine, serine and threonine metabolism	50
	Lysine degradation	25	Lysine degradation	0
	Tryptophan metabolism	37	Tyrosine metabolism	33
	Tyrosine metabolism	51	Tyrosine metabolism	0
	Valine, leucine and isoleucine degradation	30	Valine, leucine and isoleucine degradation	41
	Amino sugar and nucleotide sugar metabolism	74	Amino sugar and nucleotide sugar metabolism	86
	Citrate cycle (TCA cycle)	30	Citrate cycle (TCA cycle)	31
	Glycolysis / Gluconeogenesis	37	Glycolysis / Gluconeogenesis	35
	Pyruvate metabolism	38	Pyruvate metabolism	32
	Starch and sucrose metabolism	26	Starch and sucrose metabolism	37

	Oxidative phosphorylation	138	Oxidative phosphorylation	112
	2-Oxocarboxylic acid metabolism	32	2-Oxocarboxylic acid metabolism	36
	Biosynthesis of amino acids	111	Biosynthesis of amino acids	123
	Carbon metabolism	89	Carbon metabolism	107
	Fatty acid metabolism	48	Fatty acid metabolism	47
	N-Glycan biosynthesis	37	N-Glycan biosynthesis	47
	Other types of O-glycan biosynthesis	26	Other types of O-glycan biosynthesis	0
	Various types of N-glycan biosynthesis	32	Various types of N-glycan biosynthesis	44
	Fatty acid biosynthesis	24	Fatty acid degradation	33
	Fatty acid degradation	35	Fatty acid degradation	0
	Glycerolipid metabolism	40	Glycerolipid metabolism	28
	Glycerophospholip id metabolism	39	Glycerophospholip id metabolism	47
	Riboflavin metabolism	40	Riboflavin metabolism	32
	Glutathione metabolism	51	Glutathione metabolism	37
	Purine metabolism	109	Purine metabolism	99
	Pyrimidine metabolism	88	Pyrimidine metabolism	90
Genetic Information Processing	Proteasome	34	Proteasome	36
	Protein export	0	Protein export	27
	Protein processing in endoplasmic reticulum	114	Protein processing in endoplasmic reticulum	133
	RNA degradation	56	RNA degradation	67
	SNARE interactions in vesicular transport	0	SNARE interactions in vesicular transport	30

	Ubiquitin mediated proteolysis	49	Ubiquitin mediated proteolysis	71
	DNA replication	40	DNA replication	39
	Mismatch repair	0	Mismatch repair	28
	Nucleotide excision repair	37	Nucleotide excision repair	55
	Basal transcription factors	31	Basal transcription factors	39
	RNA polymerase	33	RNA polymerase	41
	Spliceosome	94	Spliceosome	109
	Aminoacyl-tRNA biosynthesis	45	Aminoacyl-tRNA biosynthesis	53
	RNA transport	90	RNA transport	123
	Ribosome	118	Ribosome	150
	Ribosome biogenesis in eukaryotes	73	Ribosome biogenesis in eukaryotes	103
	mRNA surveillance pathway	48	mRNA surveillance pathway	66
	Base excision repair	27	Base excision repair	0
Total		3742		4321

Table S11 The KOG classes in the *C coronatus* vs *C. obscurus*

Class Name		*C. coronatus*	*C. obscurus*
RNA processing and modification	A	248	329
Chromatin structure and dynamics	B	105	113
Energy production and conversion	C	391	339
Cell cycle control, cell division, chromosome partitioning	D	194	214
Amino acid transport and metabolism	E	369	349
Nucleotide transport and metabolism	F	81	82
Carbohydrate transport and metabolism	G	251	286
Coenzyme transport and metabolism	H	90	107
Lipid transport and metabolism	I	393	442
Translation, ribosomal structure and biogenesis	J	343	424
Transcription	K	332	370
Replication, recombination and repair	L	177	209
Cell wall/membrane/envelope biogenesis	M	74	115
Cell motility	N	5	6
Posttranslational modification, protein turnover, chaperones	O	660	789
Inorganic ion transport and metabolism	P	188	198
Secondary metabolites biosynthesis, transport and catabolism	Q	262	237
General function prediction only	R	915	1046
Function unknown	S	376	462
Signal transduction mechanisms	T	378	564
Intracellular trafficking, secretion, and vesicular transport	U	318	403
Defense mechanisms	V	67	51
Extracellular structures	W	10	20
Nuclear structure	Y	26	43
Cytoskeleton	Z	113	141
total		5571	6465

Table S12 The different number of Carbohydrate enzyme family genes in *C. coronatus* and *C. obscurus*.

Families of Carbohydrate-Active enZYmes	*C. coronatus*	*C. obscurus*
GH13	2	5
GH132	3	4
GH133	2	2
GH15	2	1
GH16	17	18
GH17	6	7
GH18	21	36
GH20	6	4
GH25	3	1
GH3	3	4
GH31	5	9
GH37	2	2
GH38	2	3
GH47	8	10
GH5	8	9
GH63	1	0
GH72	6	7
GH74	3	1
GH81	1	1
GH24	0	3
GH109	0	2
GT1	46	35
GT11	2	0
GT14	1	0
GT15	17	6
GT2	18	19
GT20	2	3
GT22	3	1
GT24	2	1
GT28	1	3
GT3	1	1
GT32	10	13
GT33	2	1
GT39	4	7
GT4	4	0
GT41	1	1
GT48	2	8
GT50	1	5

GT57	2	2
GT58	1	1
GT59	1	0
GT65	2	0
GT66	1	1
GT68	5	0
GT69	1	0
GT71	8	0
GT76	1	0
GT78	2	0
GT21	0	3
GT35	0	1
GT76	0	2
GT8	0	1
CE1	11	19
CE10	57	33
CE12	2	1
CE14	1	3
CE16	7	4
CE3	1	1
CE4	4	11
CE5	1	0
CE9	1	1
CBM18	5	7
CBM19	5	11
CBM21	1	3
CBM32	2	1
CBM43	5	6
CBM48	1	3
CBM50	2	0
CBM63	8	5
CBM68	1	0
CBM38	0	1
AA11	32	25
AA2	2	2
AA3	12	13
AA4	2	1
AA6	14	12
AA7	9	2
AA9	1	0
AA1	0	1
PL22	1	1

Table S13 T20 Secreted protein of Pfam annotation

Functions	*C. coronatus*	*C. obscurus*
Common central domain of tyrosinase	55	24
Subtilase family	33	33
Lipase (class 3)	24	45
Cysteine-rich secretory protein family	23	15
Trypsin	23	45
Small secreted domain (DUF320)	22	0
Zinc carboxypeptidase	22	6
FAD binding domain	14	2
Peptidase family M28	12	0
Fungalysin metallopeptidase (M36)	11	0
Glycosyl hydrolases family 16	10	8
GMC oxidoreductase	9	13
Calcineurin-like phosphoesterase	8	6
NAD(P)-binding Rossmann-like domain	8	0
Ribonuclease T2 family	8	1
Peptidase inhibitor I78 family	7	0
SMP-30/Gluconolaconase/LRE-like region	6	1
Thioredoxin	6	0
Type I phosphodiesterase / nucleotide pyrophosphatase	6	0
Alkaline phosphatase	5	3

Table S14 Serine Protease Genes Number in *C. coronatus* and *C. obscurus*.

Families	*C. coronatus*	*C. obscurus*
S1A	90	71
S8	60	54
S9	18	13
S10	12	4
S16	2	1
S26	2	4
S28	8	15
S54	4	5
S59	1	1
S66	1	0

Table S15 Metallopeptidases categories of *C. coronatus*

Pfam ID	Families	*C. coronatus*	*C. obscurus*
PF04389.12	M28	34	25
PF02128.10	M36	14	1
PF01546.23	M20/M25/M40	14	5
PF09471.5	M64	9	13
PF00557.19	M24	9	7
PF01433.15	M1	8	5
PF01435.13	M48	7	3
PF02127.10	M18	4	1
PF01432.15	M3	2	9
PF01434.13	M41	2	1
PF09768.4	M76	2	3
PF02163.17	M50	1	2
PF00675.15	M16	1	3

Table S16 Transcription factor categories of *C. coronatus*

Pfam annotation	*Conidiobolus coronatus*	*Conidiobolus obscurus*
Fungal specific transcription factor domain	67	25
bZIP transcription factor	11	15
Histone-like transcription factor (CBF/NF-Y) and archaeal histone	6	5
Transcription factor S-II (TFIIS)	5	4
Transcription factor TFIIB repeat	3	2
SRF-type transcription factor (DNA-binding and dimerisation domain)	2	4
STE like transcription factor	2	2
Transcription initiation factor IID, 18kD subunit	2	4
Apoptosis-antagonizing transcription factor, C-terminal	1	1
CCAAT-binding transcription factor (CBF-B/NF-YA) subunit B	1	1
CP2 transcription factor	1	1
Early transcription elongation factor of RNA pol II, NGN section	1	1
RNA polymerase I specific transcription initiation factor RRN3	1	1
RNA polymerase I-specific transcription initiation factor Rrn7	1	0
RNA polymerase II transcription factor SIII (Elongin) subunit A	1	0
RNA polymerase III transcription factor (TF) IIIC subunit	1	1
Transcription elongation factor Elf1 like	1	0
Transcription factor AFT	1	0
Transcription factor e(y)2	1	1
Transcription factor IIA, alpha/beta subunit	1	1
Transcription factor IIIC subunit delta N-term	1	0
Transcription factor Opi1	1	1
Transcription factor Pcc1	1	0
Transcription factor S-II (TFIIS), central domain	1	1
Transcription factor subunit Med10 of Mediator complex	1	1
Transcription factor Tfb2	1	1
Transcription factor Tfb4	1	1
Transcription factor TFIID (or TATA-binding protein, TBP)	1	1
Transcription factor TFIID complex subunit 8 C-term	1	0
Transcription factor/nuclear export subunit protein 2	1	1
Transcription initiation factor IIA, gamma subunit, helical domain	1	0
Transcription initiation factor IID, 31kD subunit	1	1

Transcription initiation factor IIF, beta subunit	1	0
Transcription initiation factor TFIID 23-30kDa subunit	1	1
Transcription initiation factor TFIID subunit A	1	1
Total	125	79

Table S17 Swissprot-annotated functions of genes with fungal specific transcription factor domain

#GeneID	Swissprot_annotation
EVM0G086700.1	Nitrogen assimilation transcription factor nirA GN=nirA OS=Emericella nidulans (strain FGSC A4 / ATCC 38163 / CBS 112.46 / NRRL PE=3 SV=1
EVM0G034940.1	Protein STB5 GN=STB5 OS=Saccharomyces cerevisiae (strain ATCC 204508 / S288c) PE=1 SV=1
EVM0G052800.1	Arabinolytic transcriptional activator araR OS=Emericella nidulans (strain FGSC A4 / ATCC 38163 / CBS 112.46 / NRRL PE=3 SV=1
EVM0G011010.1	Nitrogen assimilation transcription factor nit-4 GN=nit-4 OS=Neurospora crassa (strain ATCC 24698 / 74-OR23-1A / CBS 708.71 / DSM PE=3 SV=2
EVM0G057300.1	Cutinase transcription factor 1 alpha GN=CTF1-ALPHA OS=Fusarium solani subsp. pisi (Nectria haematococca) PE=2 SV=1
EVM0G037960.1	Cutinase transcription factor 1 alpha GN=CTF1-ALPHA OS=Fusarium solani subsp. pisi (Nectria haematococca) PE=2 SV=1
EVM0G058570.1	Nitrogen assimilation transcription factor nirA GN=nirA OS=Emericella nidulans (strain FGSC A4 / ATCC 38163 / CBS 112.46 / NRRL PE=3 SV=1
EVM0G086670.1	Nitrogen assimilation transcription factor nirA GN=nirA OS=Emericella nidulans (strain FGSC A4 / ATCC 38163 / CBS 112.46 / NRRL PE=3 SV=1
EVM0G105340.1	Transcriptional activator protein DAL81 GN=DAL81 OS=Saccharomyces cerevisiae (strain ATCC 204508 / S288c) PE=1 SV=3
EVM0G108870.1	Nitrogen assimilation transcription factor nit-4 GN=nit-4 OS=Neurospora crassa (strain ATCC 24698 / 74-OR23-1A / CBS 708.71 / DSM PE=3 SV=2
EVM0G102680.1	Regulator of drug sensitivity 1 GN=RDS1 OS=Saccharomyces cerevisiae (strain ATCC 204508 / S288c) PE=1 SV=1

Table S18 Gene clusters for secondary metabolite biosynthesis in *C. coronatus* genome

#Gene_cluster	scaffold_id	Start	End	Length(bp)	Annotation
r3c1	Contig00003	31237	68888	37652	NRPS-like_enzyme
r3c2	Contig00003	211080	243914	32835	NRPS-like_enzyme
r3c3	Contig00003	269889	346484	76596	NRPS-like_enzyme
r9c1	Contig00009	562937	611295	48359	NRPS-like_enzyme
r15c1	Contig00015	662147	721943	59797	NRPS
r16c1	Contig00016	1565333	1586891	21559	Terpene
r19c1	Contig00019	693516	714752	21237	Terpene
r21c1	Contig00021	246085	293491	47407	NRPS
r22c1	Contig00022	225605	269921	44317	NRPS-like_enzyme
r32c1	Contig00032	271853	320352	48500	NRPS-like_enzyme
r34c1	Contig00034	833658	876792	43135	NRPS-like_enzyme

Table S19 Molecular Mechanisms Underlying *Conidiobolus coronatus* Broad Host Adaptability Across Infection Stages

Infection Stage	Key Molecular Components	Functional Role	Evidence in *C. coronatus*
**1. Host Recognition & Signal Transduction**	**GPCRs** (125 genes)	- Detect host-specific signals (e.g., isoflavones, hydrophobicity)<br>- Activate downstream signaling pathways (MAPK, cAMP-PKA, Ca^{2+}) to trigger infection structures	- Expansion vs. specialists (*M. acridum*: 37 GPCRs) [51][55]<br>- Diverse motif architectures enable cross-host sensing [54]
	**Transcription Factors** (e.g., TUP1, prz1, GIS1)	- Reprogram gene expression for host adaptation<br>- Regulate calcium homeostasis, virulence gene repression, nutrient assimilation	- Putative roles in germination (prz1) and immune evasion (TUP1) [57][58]<br>- lncRNAs implicated in virulence attenuation (*C. obscurus*) [61]
**2. Cuticle Penetration**	**Cuticle-Degrading Enzymes (CDEs)**:<br>- Serine proteases (S1: 90 genes; S8: 60 genes)<br>- Chitinases (GH18 family)<br>- Lipases	- Degrade protein-chitin-lipid matrix<br>- Synergistic hydrolysis: Proteases + chitinases breach cuticle; Lipases target epicuticular waxes<br>- CE10 esterases enhance chitinase activity	- Gene expansion vs. specialists (*C. obscurus*)<br>- Individual proteases contribute 19–29% virulence (*B. bassiana*) [64]<br>- CAZyme repertoire: 430 genes across 6 modules
**3. Hemocoel Colonization & Immune Evasion**	**Effector Molecules**:<br>- Cytochrome P450s (127 genes; e.g., CYP51)<br>- Tyrosinases	- **P450s**: Detoxify host compounds; synthesize ergosterol for membrane integrity [65]<br>- **Tyrosinases**: Produce melanin to resist ROS/host enzymes [66]	- P450 expansion (*C. obscurus*: 124 genes)<br>- CRISP count higher than specialists (*C. obscurus*: 22 genes)<br>- Metabolomics confirms

Infection Stage	Key Molecular Components	Functional Role	Evidence in *C. coronatus*
	(55 genes)<br>- CRISPs (35 genes)<br>- Non-ribosomal peptides (e.g., coronatin-1/2)	- **CRISPs**: Disrupt hemocyte function via ion channel modulation [67][68]<br>- **Coronatins**: Induce hemocyte apoptosis [69][70]	terpenoid/alkaloid production [75]
**4. Host-Specific Adaptation & Toxin Deployment**	**Horizontally Acquired Toxins** (e.g., CytCo-like genes, destruxin analogs)<br>**Broad-Spectrum Metabolites** (e.g., sesquiterpenes, harman/norharman)	- Target conserved host pathways (e.g., calcium homeostasis, nerve-muscle ion channels)<br>- Overcome genotype-specific immune variations via "mixed outcome" phenotypes	- 240 PHI genes linked to variable infection outcomes<br>- Alkaloids active against Lepidoptera [75]<br>- Terpenoid clusters suggest insecticidal/plant growth effects

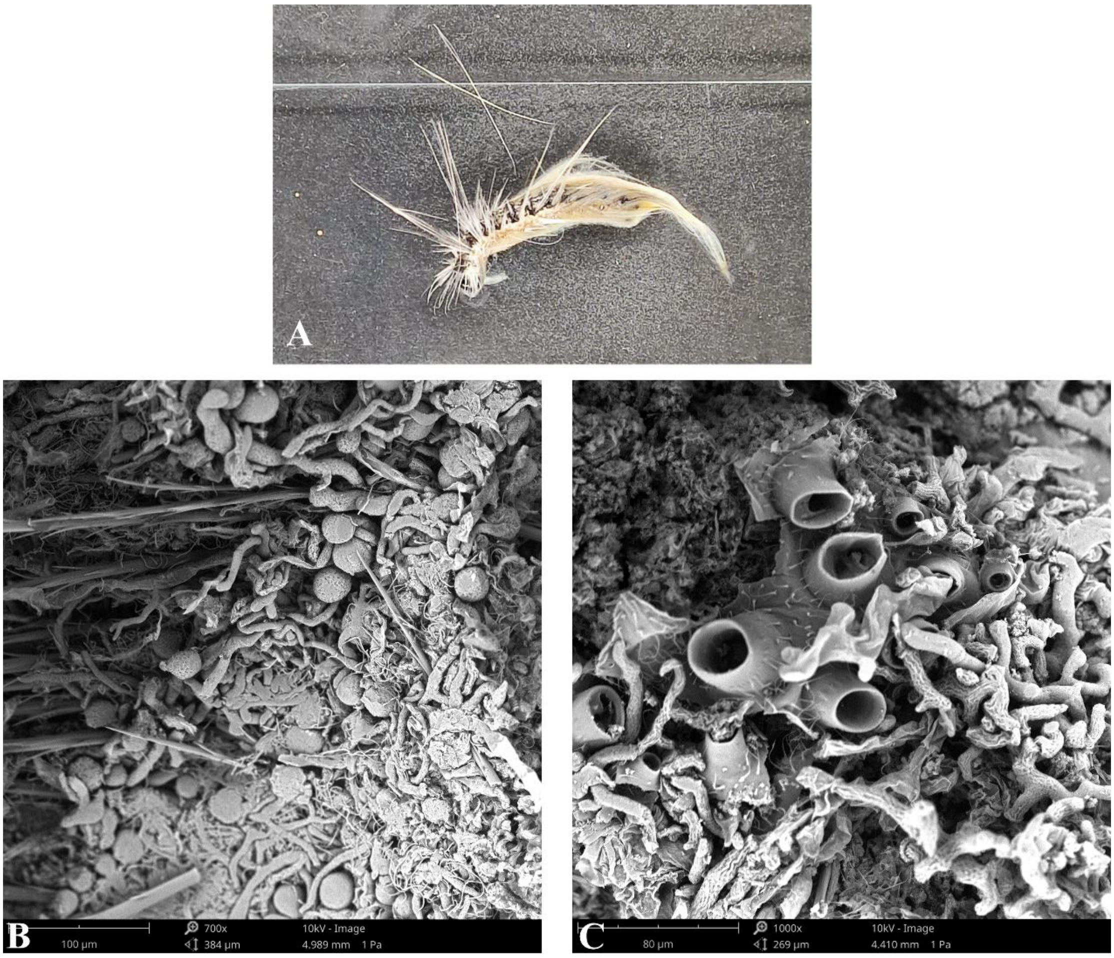

Figure S1. Images of *Hyphantria cunea* larvae infected by *Conidiobolus coronatus* after conidial inoculation. (A) A white spore halo surrounding a cadaver of infected larvae following overnight incubation under saturated humidity. (B and C) Scanning electron microscopy (SEM) images of mycotized *H. cunea*.

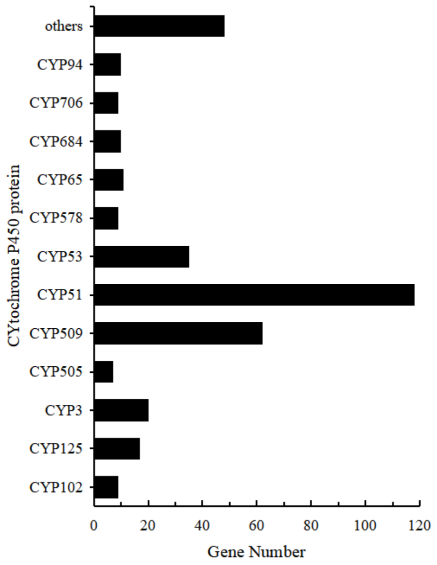

Figure S2.P450 family classification statistics of *C. coronatus*

Cytochrome P450 (CYP) enzymes play diverse and critical roles in insect-pathogenic fungi, including degradation of insect cuticles, interference with host immunity, production of toxic metabolites, and detoxification. Through annotation against the CYPED (CYtochrome P450 Engineering Database), 365 P450-related genes were identified. The CYP51 family was the most abundant (118 genes), primarily involved in maintaining cell membrane integrity by catalyzing the 1α-demethylation reaction in the ergosterol biosynthesis pathway.

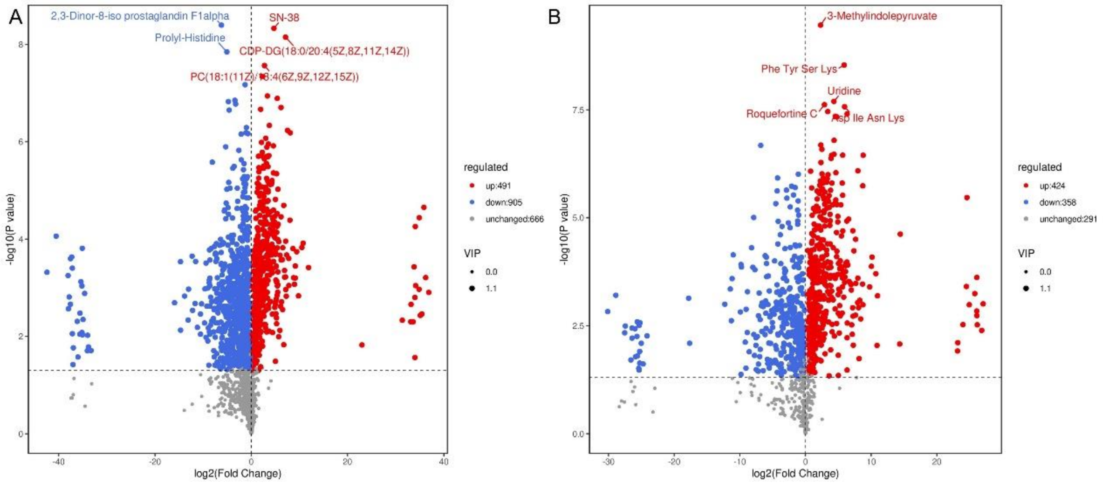

Figure S3. Volcano plots of differentially abundant metabolites between *C. coronatus* and *C. obscurus*. (A) positive ion mode, (B) negative ion mode. The x-axis represents $\log_2$ fold change, and the y-axis represents $\log_{10}$ *P*-values. Metabolites are color-coded: blue points represent downregulated metabolites, whereas red points represent upregulated metabolites in *C. coronatus*. The circle size of each data point represents the variable importance in projection (VIP) value calculated using the orthogonal partial least squares discriminant analysis (OPLS-DA) model, where large circles correspond to high VIP values, indicating reliable differential expression of metabolites. The top five metabolites are annotated.
